# Supplementary material for: Splice-Junction-Based Mapping of Alternative Isoforms in the Human Proteome
Source: Cell Rep. Author manuscript; Available in PMC 2020 Jan 15. (PMC6961840; doi:10.1016/j.celrep.2019.11.026)
Supplement: 2 [file NIHMS1546469-supplement-2.zip › DF1/PXD010154/Esophagus/Esophagus_15_TUBG1_EADDEHYIPR.pdf]

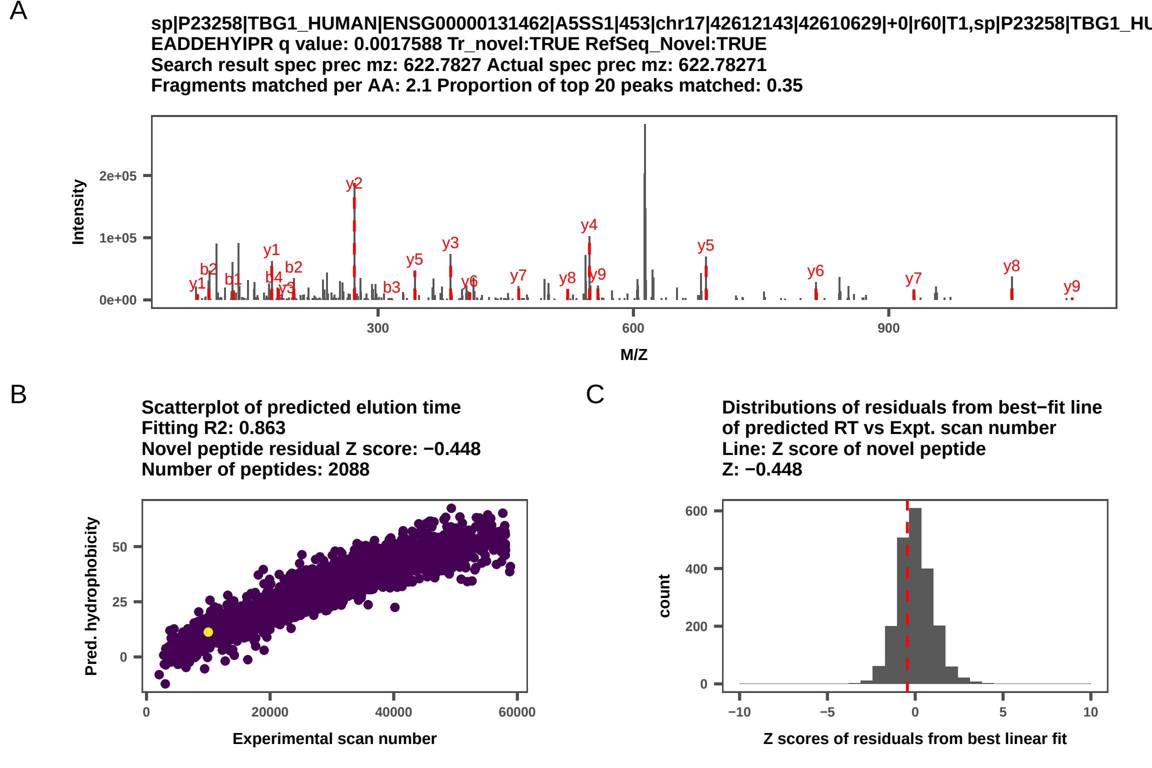
A
sp|P23258|TBG1_HUMAN|ENSG00000131462|A5SS1|453|chr17|42612143|42610629|+0|r60|T1,sp|P23258|TBG1_HU
EADDEHYIPR q value: 0.0017588 Tr_novel:TRUE RefSeq_Novel:TRUE
Search result spec prec mz: 622.7827 Actual spec prec mz: 622.78271
Fragments matched per AA: 2.1 Proportion of top 20 peaks matched: 0.35
Intensity
2e+05
1e+05
0e+00
y1
b2
b1
y1
b4
y3
b2
y2
b3
y5
y3
y6
y7
y8
y4
y9
y5
y6
y7
y8
y9
300
600
900
M/Z
B
Scatterplot of predicted elution time
Fitting R2: 0.863
Novel peptide residual Z score: −0.448
Number of peptides: 2088
Pred. hydrophobicity
50
25
0
0
20000
40000
60000
Experimental scan number
C
Distributions of residuals from best−fit line
of predicted RT vs Expt. scan number
Line: Z score of novel peptide
Z: −0.448
count
600
400
200
0
−10
−5
0
5
10
Z scores of residuals from best linear fit
